# Genetic and Anthropometric Interplay: How Waist-to-Hip Ratio Modulates LDL-c Levels in Mexican Population

**DOI:** 10.3390/nu16193402

**Published:** 2024-10-08

**Authors:** César Hernández-Guerrero, Erika Arenas, Jaime García-Mena, Edgar J. Mendivil, Omar Ramos-Lopez, Graciela Teruel

**Affiliations:** 1 Departamento de Salud, Universidad Iberoamericana Ciudad de México, Mexico City 01219, Mexico; edgar.mendivil@ibero.mx; 2 Department of Sociology, University of California, Santa Barbara, CA 93106, USA; earenas@soc.ucsb.edu; 3 Departamento de Genética y Biología Molecular, Centro de Investigación y de Estudios Avanzados del Instituto Politécnico Nacional, Mexico City 07360, Mexico; jgmena@cinvestav.mx; 4 Medicine & Psychology Faculty, Autonomous University of Baja California, Tijuana 22390, Mexico; oscar.omar.ramos.lopez@uabc.edu.mx; 5 División de Estudios Sociales, Universidad Iberoamericana Ciudad de México, Mexico City 01219, Mexico

**Keywords:** obesity, SNP, *ABCA1*, *ADIPOQ*, *FTO*, *GRB14*, *LEPR*, genetic risk score

## Abstract

Background/Objectives: Genetic factors contribute to the physiopathology of obesity and its comorbidities. This study aimed to investigate the association of the SNPs *ABCA1* (rs9282541), *ADIPOQ* (rs2241766), *FTO* (rs9939609), *GRB14* (rs10195252), and *LEPR* (rs1805134) with various clinical, anthropometric, and biochemical variables. Methods: The study included 396 Mexican mestizo individuals with obesity and 142 individuals with normal weight. Biochemical markers were evaluated from peripheral blood samples, and SNP genotyping was performed using PCR with TaqMan probes. A genetic risk score (GRS) was computed using an additive model. Results: No significant associations were found between the SNPs *ABCA1*, *ADIPOQ*, *FTO*, and *LEPR* with obesity. However, the T allele of the *GRB14* SNP was significantly associated with obesity (χ^2^ = 5.93, *p* = 0.01; OR = 1.52; 95% CI: 1.08–2.12). A multivariate linear regression model (adjusted R-squared: 0.1253; *p* < 0.001) predicting LDL-c levels among all participants (n = 538) identified significant (*p* < 0.05) beta coefficients for several anthropometric and biochemical variables, as well as for the GRS. Additionally, the interaction between the GRS and the waist-to-hip ratio (WHR) showed a negative beta coefficient (BC = −26.5307; *p* = 0.014). Participants with a WHR < 0.839 showed no effect of GRS on LDL-c concentration, while those with a WHR > 0.839 exhibited a greater effect of GRS (~9) at lower LDL-c concentrations (~50 mg/dL) and a lesser effect of GRS (~7) at higher LDL-c concentrations (~250 mg/dL). Conclusions: A significant interaction between genetics and WHR influences LDL-c in Mexicans, which may contribute to the prevention and clinical management of dyslipidemia and cardiovascular disease.

## 1. Introduction

Obesity is a multifactorial chronic disease characterized by abnormal or excessive fat accumulation, which is associated with several comorbidities [1]. A body mass index (BMI) exceeding 30 kg/m^2^ defines obesity as a known precursor to chronic conditions, including cerebrovascular disease, type 2 diabetes, dyslipidemia, metabolic syndrome, and cancer [2]. Approximately 4.72 million deaths annually are attributable to obesity and its comorbidities [3]. In Mexico, the public health concern is evident, with 33.3% of school-age children, 35% of adolescents, and over 75% of adults experiencing obesity or overweight [4,5]. Treating obesity-related conditions costs around 2 trillion dollars globally, representing 2.8% of the global gross domestic product [6].

Obesity is a multifactorial phenomenon that develops over time due to the interaction of several factors, such as the energy imbalance between intake and expenditure, which is crucial in the obesogenic process. Over the last fifty years, various factors have contributed to the rise of an obesogenic environment, promoting the development of obesity. These include the widespread availability of inexpensive large portions of highly processed energy-dense foods high in saturated fats and sugars. Additionally, people are facing a reduction in their time for physical activity due to long work hours and lengthy commutes to work and study locations, significantly decreasing the opportunities for physical activity and increasing overall sedentary behavior. On the other hand, it has been identified that not all individuals who exhibit an imbalance in energy intake and expenditure develop obesity, highlighting the significant role of another factor as the genetic background in this phenomenon [7,8,9]. Hence, given the health and socioeconomic impact of obesity and its comorbidities, understanding the relationship of factors influencing their onset and development is crucial.

One of the major comorbidities associated with obesity is cerebrovascular disease, a group of pathologies that account for approximately 30% of deaths worldwide, both in developing and developed countries. The leading causes of death from cerebrovascular disease are coronary heart disease and stroke. These conditions are strongly linked to the presence of hypertension and atherosclerosis. Atherosclerosis is characterized by fat accumulation within the arterial wall at specific sites, where oxidative, inflammatory, and necrotic processes occur, triggering the development of atherosclerotic plaques and the consequent narrowing of the blood vessel lumen. This narrowing reduces blood flow to organs such as the heart and brain. Atheroma ruptures can result in a thrombus that blocks blood circulation, potentially triggering an acute ischemic vascular event [10].

The formation of atherosclerotic plaques typically begins with an alteration in the vascular endothelium, leading to the infiltration of low-density lipoprotein cholesterol (LDL-c) and, subsequently, leukocytes, such as lymphocytes and monocytes. As the permeability of the vessel wall increases, the circulation of LDL-c passes through the vascular endothelium into the intima. Once oxidized or enzymatically glycated, the LDL-c can no longer be recognized by their specific receptors and accumulate in the extracellular space. This accumulation triggers a local inflammatory response, inducing the activation of lymphocytes and monocytes, which differentiate into macrophages. These macrophages then phagocytize the oxidized LDL-c deposits through their scavenger receptors, promoting a chronic pro-inflammatory state. The buildup of oxidized LDL-c within macrophages results in the formation of foam cells, a hallmark of the initial phases of atherosclerotic plaque formation. Poor dietary habits, characterized by an excessive consumption of highly caloric foods rich in fats and sugars, increase total cholesterol, LDL-c, and triglycerides. When these molecules remain chronically elevated in an individual, they tend to accumulate, manifesting as an increase in the body fat percentage, particularly in visceral fat. Visceral fat is implicated explicitly in promoting the chronic pro-inflammatory state observed in individuals with obesity, a pro-inflammatory process that enhances systemic atherogenic processes [10,11]. 

From this perspective, it has been identified that elevated LDL-c increases the risk of developing cardiovascular disease (CVD), with the risk increasing proportionally as the concentration and duration of elevated plasma LDL-c levels rise [12,13]. Therefore, implementing strategies aimed at managing dyslipidemias, particularly LDL-c, is of particular relevance to reduce the incidence of CVD in the population.

On the other hand, in the development of the obesogenic process and its main comorbidities, the genetic background of each individual plays an active role. In this regard, association studies using single nucleotide polymorphisms (SNPs) as markers have identified essential genes in obesity. A 2018 meta-analysis of genome-wide association studies (GWAS) in Europe identified 941 loci associated with BMI, while a 2017 global report detailed 225 SNPs linked to obesity [14,15]. The genetic background of each individual and population plays a prominent role in the onset and development of obesity and its comorbidities, as it has been reported that 50–70% of the variation in BMI is determined by genetics. This genetic component actively participates in several aspects related to BMI, such as the efficiency with which the digestive system assimilates nutrients from dietary sources, how effectively food is used as a source of energy or stored as fat, how much hunger or satiety a person feels when eating, and how the body processes carbohydrates, proteins, and lipids. All these characteristics are regulated by a specific set of genes which, through dynamic gene–environment interactions, influence the development of obesity and its comorbidities [16].

In the Mexican population, significant genes include *FTO*, *ABCA1*, *LEPR*, *GRB14*, and *ADIPOQ*, which are associated with BMI, adipogenesis, insulin signaling, cholesterol homeostasis, and adipokine regulation. For instance, the *FTO* SNP is linked to increased BMI and systemic inflammation; *ADIPOQ* and *GRB14* SNPs are associated with metabolic syndrome and increased adiposity; the *ABCA1* variant is related to low HDL-c levels; and *LEPR* may contribute to morbid obesity in adults [17,18,19,20,21,22]. 

Within the context of personalized medicine, these genetic profiles and their interaction with environmental or phenotypic factors may explain the individual variability in lipid metabolism and the related cardiovascular risk, as well as facilitate the implementation of precision strategies to achieve a more significant impact on cardiovascular prevention [23,24]. This approach could complement current interventions and health programs aimed at promoting cardiovascular health.

This study analyzes the prevalence of several SNPs in a Mexican mestizo population with individuals with normal weight and obesity and their relationship with the levels of LDL-c and different anthropometric variables to identify markers to improve the diagnosis and prognosis of principal comorbidities like CVD, a principal cause of deaths in our country and worldwide.

## 2. Materials and Methods

### 2.1. Study Population

The study included 254 individuals with obesity and 142 individuals with normal weight from Mexico City, aged from 19 to 65 years. The participants who sought nutritional counseling at the Nutrition Clinic of Universidad Iberoamericana in Mexico City were invited to enroll in the study. During their first visit to the clinic, participants completed a questionnaire covering their medical history, physical activity, and diet. The university’s Ethics Committee reviewed and approved the study, and all participants provided informed consent before participating.

Individuals with obesity (BMI ≥ 30) were eligible for inclusion if they did not have autoimmune diseases, cancer, eating disorders, or pregnancy, if they were not breastfeeding, and if they had not experienced any acute illness and were not taking lipid-lowering drugs in the previous six months. The same criteria were applied to individuals with normal weight (BMI = 18.5–24.9), who were required to be clinically healthy at the time of inclusion. All participants were of Mexican mestizo origin, with both parents and grandparents born in Mexico.

The study was classified as a minimal risk to participants’ health and offered several benefits. Participants received a thorough explanation of the project’s objectives, scope, and goals, as well as the benefits of participation. It was emphasized that participation was voluntary and that it would not affect the quality of care they received if they chose not to participate. Information about the confidentiality of their personal data was provided, and participants were informed of their right to withdraw from the study at any time. They could also request additional information about the project at any stage.

### 2.2. Blood Sampling

A peripheral venous blood sample was collected from fasting individuals, and DNA was extracted using the DNAzol method. After collecting biological material, the sample was stored at −60 °C. The quantity and purity of the DNA were assessed using the 260/280 nm ratio derived from spectrophotometric measurements, with a purity range of 1.8–2.0 deemed acceptable for subsequent genotyping experiments.

### 2.3. Biochemical Assays

The total cholesterol, triglycerides, HDL-c, LDL-c, and glucose concentrations were determined for all participants using the DRI-CHEM NX500i equipment (Fujifilm, Tokyo, Japan), following the manufacturer’s directions.

### 2.4. SNP Genotyping

Allelic discrimination was conducted using the RT-PCR technique on the StepOnePlus™ system for all study participants. Each DNA sample was analyzed using Taqman probes (Applied Biosystems™, Thermo Fisher Scientific, Waltham, MA, USA) for the following polymorphisms: *ABCA1* rs9282541 (C_11720861_10, G>A), *GRB14* rs10195252 (C_121021_10, T>C), *FTO* rs9939609 (C_30090620_10, T>A), *ADIPOQ* rs2241766 (C_26426077_10, T>G), and *LEPR* rs1805134 (C_11874781_10, T>C). The assays were conducted using 48-well plates, and samples were analyzed in duplicate. Manufacturer recommendations and guidelines were strictly followed, with appropriate controls employed in all experiments to ensure accurate genotyping. A genetic risk score (GRS) was computed using an additive model, as previously reported [25]. The number of high-risk alleles at each locus was summed; thus, each unit increase in the GRS corresponded to one additional risk allele.

### 2.5. Statistical Analysis

Statistical analyses were performed using SigmaPlot 12.0 software. The association between the frequency of each genetic variant and the presence of obesity was examined using the χ^2^ test. Multivariate correlation analyses were conducted using STATA software (version 12). Comparisons of variables between individuals with obesity and those with normal weight were performed using the Student’s t-test or the Mann–Whitney test, as appropriate. A statistically significant difference was recognized when the *p* value was less than 0.05. Hardy–Weinberg equilibrium was calculated for the genes studied.

## 3. Results

### 3.1. Demographic Characteristics and Clinical Features

The study included 393 participants, categorized into two groups: normal-weight individuals (142 participants) and individuals with obesity (254 participants). Table 1 presents each group’s clinical, biochemical, and anthropometric values. Statistically significant differences were observed in most of the analyzed variables, except for total cholesterol and LDL-c.

### 3.2. Hardy–Weinberg Equilibrium

The Hardy–Weinberg equilibrium test was conducted for the five genes examined in the study. For four of the five genes, the Hardy–Weinberg equilibrium values were not statistically significant among all participants (Table 2). However, a Hardy–Weinberg equilibrium value of *p* < 0.001 was observed for the *FTO* SNP.

### 3.3. Genotypic and Allelic Frequencies in Study Groups

The allelic and genotypic frequencies of the five analyzed SNPs in participants from the obesity and normal-weight groups are presented in Table 3 and Table 4. Two types of analyses were conducted for each polymorphism. The first, an allele-based analysis, involved grouping participants based on the presence or absence of the risk allele. The second, a carrier-based analysis, considered genotypes (homozygotes plus heterozygotes) carrying the risk allele.

For four of the five studied SNPs, no statistically significant differences were observed when comparing the frequency of individuals with obesity to those in the normal-weight group. However, the SNP *GRB14* (rs10195252) showed a statistically significant difference in carrier frequency (*p* = 0.02), being 0.37 in the obesity group compared to 0.48 in the normal-weight group. Additionally, a statistically significant difference was observed in its risk allelic frequency (*p* = 0.01), being 0.21 in the obesity group versus 0.29 in the normal-weight group.

### 3.4. Characteristics of Participants Stratified by LDL-c

Table 5 presents the values of the anthropometric and metabolic variables of the study population: n = 396 (female n = 306 (77.2%), male n = 64 (22.8%)), divided into groups based on LDL-c levels below 130 mg/dL (n = 366) and above 130 mg/dL (n = 30). A statistically significant difference between the two stratified LDL-c groups was observed only in fasting glucose concentration, total cholesterol, and HDL-c. Even though 77% of participants were women, a multivariate linear regression model predicting LDL-c levels (Table 6) was adjusted by female gender (*p* = 0.125).

### 3.5. Effect of GRS and WHR on LDL-c Prediction

Table 6 presents the multivariate linear regression model values to predict LDL-c. Statistically significant associations were identified with biochemical markers, anthropometric variables, age, the GRS, and the interaction between the GRS and the WHR. The adjusted R-squared value of the regression model demonstrated a very robust statistical significance (*p* < 0.001).

**Figure 1:**
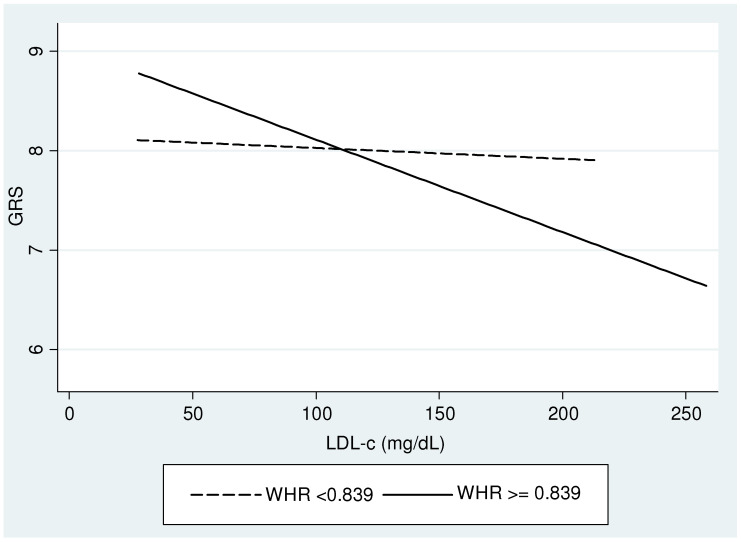
Interaction between GRS and WHR regarding LDL-c. Analyses were adjusted by age and gender.

Figure 1 illustrates the interaction between the GRS and the waist-to-hip ratio in the study participants, divided into two groups based on a threshold of 0.839. In the group with a waist-to-hip ratio below 0.839, the interaction between the studied genes and plasma LDL-c levels is minimal. The GRS remains stable at a value close to 8.0 across a range of plasma LDL-c from 50 to approximately 200 mg/dL. Conversely, a clear negative slope is observed in the group with a WHR above 0.839. The GRS starts at around 9.0 when LDL-c is near 50 mg/dL and decreases to approximately 6.5 as LDL-c exceeds 250 mg/dL.

## 4. Discussion

This study analyzed five SNPs previously associated with various metabolic diseases and obesity in Mexican participants. For the SNP *ABCA1* (rs9282541; G>A), the risk allele (A) frequency was similar between the normal-weight group (0.05) and the obesity group (0.08). These results agree with earlier research, including that of Genis-Mendoza et al. (2020), who reported a frequency of 0.11 in the obesity group [26]. Similarly, Flores-Viveros et al. (2019) and León-Reyes et al. (2023) reported frequencies of 0.12 and 0.10, respectively. Larger studies, such as Ochoa-Guzmán (2020) and Romero-Hidalgo (2012), reported frequencies of 0.12 and 0.09, respectively [27,28,29,30]. Our study found no significant association between the risk allele or genotype and obesity, a finding also reported by Velázquez-Román et al. (2021) in their study on obesity, type 2 diabetes, and metabolic syndrome [31]. Conversely, Ochoa-Guzmán identified significant associations with type 2 diabetes, insulin resistance, and low HDL-c [29], while Flores-Viveros et al. (2019) reported significant associations between the risk allele and obesity, as well as an increased risk of developing metabolic syndrome [27].

For the SNP *LEPR* (rs1805134; T>C), the risk allele (C) frequency was similar between the normal-weight group (0.16) and the obesity group (0.13). Our study did not find an association between this risk allele or genotype and obesity. The observed risk allele frequency in the obesity group (0.13) aligns with Rojano-Rodríguez et al. (2016), who found a frequency of 0.17 in individuals with morbid obesity and 0.06 in normal-weight individuals. They reported a significant association between the risk allele and morbid obesity [20]. However, Rocío Aller et al. (2023) found a risk allele frequency of 0.20 in a Caucasian population with obesity, which is slightly higher than our finding [32]. Rocío Aller also reported that carriers of the risk genotype had higher biochemical and adiposity-related values and a stronger predisposition to metabolic syndrome, which was not observed in our study [32].

For SNP *GRB14* (rs10195252; T>C), we observed a higher risk allele (C) frequency in the normal-weight group (0.29) compared to the obesity group (0.22). Miranda-Lora et al. found that the wild-type allele (T) was associated with elevated glucose, triglycerides, insulin levels, and WHR in Mexican mestizo children [33]. Peralta-Romero reported an association between the allele (T) and increased waist circumference in Mexican children with obesity [34]. Although our study did not find significant associations with the variables studied, we identified a significant association (*p* = 0.01, OR = 1.52; 95% CI 1.08–2.12) between allele T and obesity, though carriers of the risk allele did not show a significant association with obesity (*p* = 0.12).

For SNP *ADIPOQ* (rs2241766; T>G), the risk allele (G) frequency was similar between the normal-weight group (0.18) and the obesity group (0.22). Our study found no association between the risk allele or genotype and obesity. The frequency of the risk allele in individuals with obesity (0.22) is consistent with Peralta-Romero et al. (2015), who reported frequencies of 0.18 in children with obesity and 0.19 in normal-weight individuals [34]. However, a meta-analysis by Wang and et al. identified an association between the *ADIPOQ* risk allele and dyslipidemia. It reduced adiponectin and HDL-c levels and increased triglycerides, total cholesterol, and LDL-c [35]. Our study did not find significant differences in the analyzed variables between carriers and non-carriers of the risk allele.

For the *FTO* SNP (rs9939609; T>A), the risk allele (A) frequency was similar between the normal-weight group (0.32) and the obesity group (0.27). We found no significant association between the risk allele or genotype and obesity. The risk allele frequency in normal-weight individuals (0.32) was higher than Villalobos-Comparán et al. reported, which was 0.21 in the normal-weight Mexican population, similar to the 0.25 reported in type 2 diabetes cases [36]. Our findings align with those of Abadi et al. (2016), who did not find an association between the *FTO* SNP and obesity in Mexican mestizo children [37]. However, Garcia-Solís et al. (2016) and Ortega et al. (2021) identified associations between the *FTO* SNP and higher BMI, blood pressure, and hyperglycemia in Mexican populations [38,39].

The frequencies of the five SNPs studied were similar to those previously reported in different populations. Nonetheless, variations in the relationships between each SNP and obesity are probably influenced by the interplay of the genetic background of each population and environmental factors.

The frequencies of the five SNPs studied in this work were similar to those reported in previous studies on the Mexican population. However, the search for interactions between those five SNPs (GRS) and biochemical and anthropometric markers showed a strong relationship between waist-to-hip ratio values and plasma LDL-c concentration. This finding is relevant because plasma LDL-c concentration has been described as a strong marker for the development of CVD through the progression of atherosclerosis [40].

Cardiovascular disease remains a significant comorbidity associated with obesity, contributing significantly to morbidity and mortality worldwide. The development of this condition involves a complex interplay of dietary, physical activity, and genetic factors. Atherosclerosis plays a central role in the pathogenesis of cardiovascular disease, initiated by endothelial damage and chronic elevation of LDL-c levels. Maintaining LDL-c levels below 100 mg/dL is recommended, with levels between 100 mg/dL and 130 mg/dL considered acceptable for individuals without cardiovascular disease risk.

This study’s multivariate linear regression analysis to predict LDL-c levels showed robust beta coefficients and significance for anthropometric variables (BMI, WHR), biochemical variables (glucose, triglycerides), and age. The main finding of this study was that the GRS and WHR interaction analysis revealed significant beta coefficients and statistical significance. It is important to highlight that this analysis considered age and gender as covariates, given the adiposity differences between women and men [41] and the variability related to age, evidenced in the multivariate linear regression model and the interaction plot analysis. These data indicate that these covariates did not play a significant role in our results. Individuals with a WHR below 0.839 exhibited minimal impact from the genetic score. In contrast, those with a ratio above 0.839 showed a notable inverse effect of the GRS on LDL-c levels. Individuals with a GRS around 9 showed a concentration of LDL-c ~50 mg/dL, whereas those with a GRS near 6.5 showed a concentration of LDL-c ~250 mg/dL. Thus, our analyses suggest that the precise management of LDL-c as a cardiovascular disease marker should consider the genetic profile of individuals (GRS feature) together with the adiposity level, particularly in the central area. An essential issue of this finding is that WHR is a simple and accessible measurement in clinical practice, whereas the GRS was constructed by common SNPs in the Mexican population. However, further studies are needed to validate these results in other populations worldwide. Overall, these findings underscore the complex relationships between genetics and environment in developing obesity-related diseases. Identifying genetic markers specific to our population could enhance the sensitivity and specificity of prognostic studies, helping to identify individuals at higher risk for metabolic and cardiovascular diseases, which remain significant contributors to mortality globally.

The progression of obesity and its coexisting conditions, especially CVD, is a complex interaction between the environmental conditions in which a population develops and its genetic imprint. Specifically, the Mexican population strongly tends to accumulate body fat percentage and visceral adipose tissue, which is directly related to the waist-to-hip ratio (WHR). Higher visceral fat levels and, notably, higher WHR are directly associated with elevated LDL-c levels, which are, in turn, linked to an increased risk of CVD. Identifying genetic markers in the Mexican population that can be used alongside easily measured anthropometric indicators may be a powerful tool in the search for prognostic strategies in our population. These results could help identify individuals at risk of suffering from cardio- or cerebrovascular events, the leading cause of death in both the Mexican and global populations. This study shows robust associations in the regression analysis between LDL-c levels, WHR, and GRS. However, a limitation of the study is determining whether this association can be identified in other populations within our country. Due to Mexico’s high diversity in terms of ethnic background and the current presence of Amerindian populations, the interaction observed through these variables may be non-replicable.

## 5. Conclusions

A significant interaction between genetics and WHR influences LDL-c in Mexicans, which shows a complex interplay between the genetic background and anthropometry concerning lipid metabolism. These findings suggest that the genetic susceptibility to LDL-c alterations and, consequently, cardiovascular risk depend on the levels of central adiposity. This knowledge may be translated to the clinical setting for prevention purposes by calculating each person’s analyzed GRS and WHR to estimate the individual risk of LDL-c rises. Based on this information, the management of dyslipidemia and related cardiovascular diseases could be personalized based on individual genotypes and phenotypic (adiposity) characteristics.

## Figures and Tables

**Table 1:** Physical characteristics and clinical biochemical markers of study participants.

Parameter	Normal Weight (n = 142)	Obesity (n = 254)
Age (years)	26.74 ± 10.8 (23.0)	41.34 ± 12.6 * (41.0)
Weight (kg)	59.23 ± 7.5 (57.9)	96.83 ± 20.4 * (94.2)
Height (m)	1.64 ± 0.08 (1.6)	1.61 ± 0.09 * (1.5)
Body Mass Index (BMI)	21.92 ± 1.6 (21.9)	37.22 ± 6.6 * (35.5)
Waist Circumference (cm)	69.79 ± 6.4 (69.0)	104.18 ± 13.2 * (102.0)
Hip Circumference (cm)	92.36 ± 5.8 (93.0)	118.25 ± 13.8 * (115.1)
Waist-to-Hip Ratio (WHR)	0.75 ± 0.07 (0.7)	0.88 ± 0.08 * (0.8)
Abdominal Circumference (cm)	79.77 ± 7.5 (79.0)	113.12 ± 14.7 * (110.6)
Systolic Blood Pressure (mmHg)	110.35 ± 10.6 (110.0)	124.35 ± 16.4 * (120.0)
Diastolic Blood Pressure (mmHg)	73.62 ± 8.6 (70.0)	82.14 ± 11.0 * (80.0)
Glucose (mg/dL)	85.20 ± 17.1 (83.5)	99.74 ± 29.6 * (93.0)
Triglycerides (mg/dL)	97.72 ± 51.7 (87.0)	170.24 ± 79.0 * (153.5)
HDL-c (mg/dL)	48.78 ± 8.7 (49.0)	41.82 ± 9.0 * (41.0)
Total Cholesterol (mg/dL)	157.50 ± 31.6 (155.5)	164.35 ± 37.1 (159.5)
LDL-c (mg/dL)	89.16 ± 27.2 (83.9)	87.72 ± 29.6 (82.7)

Data displayed: mean ± standard deviation. Value in parenthesis: median. * *p* < 0.05; Mann–Whitney rank sum test.

**Table 2:** Hardy–Weinberg equilibrium.

SNP	Total Population (n = 396)		
	Risk Allele Frequency	Wild-Type Frequency	*p* Value
*ABCA1* (rs9282541)	A (0.07)	G (0.93)	0.70
*FTO* (rs9939609)	A (0.29)	T (0.71)	**0.001**
*GRB14* (rs10195252)	C (0.26)	T (0.74)	0.43
*ADIPOQ* (rs2241766)	G (0.2)	T (0.8)	0.64
*LEPR* (rs1805134)	C (0.14)	T (0.86)	0.21

Bold numbers indicate *p* < 0.05.

**Table 3:** Frequencies and differences among *ABCA1*, *GRB14*, and *FTO* polymorphisms identified in obesity and normal-weight groups.

	Genotype			Carriers Genotype	Risk Allele	Wild-Type Allele	X^2^	*p*	OR	CI 95%
*ABCA1* (rs9282541) G > A	GG	GA	AA	GA + AA	A	G				
Normal Weight n = 142	127 (0.89)	15 (0.11)	0 (0.0)	15 (0.11)	15 (0.05)	269 (0.95)				
OB I–III n = 254	217 (0.85)	35 (0.14)	2 (0.01)	37 (0.15)	39 (0.08)	469 (0.92)	(a) 0.95 (b) 1.28	(a) 0.32 (b) 0.25	(a) 1.44 (b) 1.49	(a) 0.76–2.73 (b) 0.80–2.75
*GRB14* (rs10195252) T > C	TT	TC	CC	TT + TC	C	T				
Normal Weight n = 142	73 (0.51)	56 (0.39)	13 (0.10)	129 (0.90)	82 (0.29)	202 (0.71)				
OB I–III n = 254	159 (0.63)	83 (0.33)	12 (0.04)	242 (0.96)	107 (0.21)	401 (0.79)	(a) 2.32 (b) 5.93	(a) 0.12 (b) 0.01	(a) 2.03 (b) 1.52	(a) 0.90–4.58 (b) 1.08–2.12
*FTO* (rs9939609) T > A	TT	TA	AA	TA + AA	A	T				
Normal Weight n = 142	72 (0.51)	49 (0.35)	21 (0.15)	70 (0.50)	91 (0.32)	193 (0.68)				
OB I–III n = 254	139 (0.55)	92 (0.36)	23 (0.09)	115 (0.45)	138 (0.27)	370 (0.73)	(a) 0.76 (b) 2.35	(a) 0.38 (b) 0.12	(a) 0.85 (b) 0.79	(a) 0.56–1.28 (b) 0.57–1.08

Data displayed: number of participants (frequency). OB I–III: participants with any type of obesity. (a) Carriers-based analysis. (b) Allele-based analysis.

**Table 4:** Frequencies and differences among *ADIPOQ* and *LEPR* polymorphisms identified in obesity and normal-weight groups.

	Genotype			Carriers Genotype	Risk Allele	Wild-Type Allele	X^2^	*p*	OR	CI 95%
*ADIPOQ* rs2241766 (T > G)	TT	TG	GG	TG + GG	G	T				
Normal Weight n = 142	98 (0.69)	38 (0.27)	6 (0.04)	44 (0.31)	50 (0.18)	234 (0.82)				
OB I–III n = 254	155 (0.61)	87 (0.34)	12 (0.05)	99 (0.39)	111 (0.22)	397 (0.78)	(a) 2.18 (b) 1.77	(a) 0.13 (b) 0.18	(a) 1.42 (b) 1.38	(a) 0.91–2.20 (b) 0.90–1.89
*LEPR* (rs1805134) T > C	TT	TC	CC	TC + CC	C	T				
Normal Weight n = 142	102 (0.72)	34 (0.24)	6 (0.04)	40 (0.28)	46 (0.16)	238 (0.84)				
OB I–III n = 254	194 (0.76)	55 (0.22)	5 (0.02)	60 (0.24)	65 (0.13)	443 (0.87)	(a) 1.25 (b) 2.04	(a) 0.26 (b) 0.15	(a) 0.78 (b) 0.75	(a) 0.49–1.25 (b) 0.50–1.14

Data displayed: number of participants (frequency). OB I–III: participants with any type of obesity. (a) Carriers-based analysis. (b) Allele-based analysis.

**Table 5:** Demographic, anthropometric, and metabolic characteristics of the total population divided by LDL-c groups.

Parameter	LDL-c < 130 mg/dL (n = 366)	LDL-c ≥ 130 mg/dL (n = 30)	*p* Value
Age (years)	35.6 ± 13.9	41.3 ± 12.2	0.031
Body Mass Index (BMI)	31.6 ± 9.3	32.2 ± 6.4	0.745
Waist Circumference (cm)	89.8 ± 23.5	92.1 ± 23.3	0.612
Hip Circumference (cm)	108.8 ± 17.5	110.4 ± 9.6	0.631
Waist-to-Hip Ratio (WHR)	0.83 ± 0.10	0.86 ± 0.11	0.215
Systolic Blood Pressure (mmHg)	121.8 ± 54.2	122.4 ± 13.7	0.948
Diastolic Blood Pressure (mmHg)	78.9 ± 11.1	80.5 ± 10.1	0.446
Glucose (mg/dL)	93.5 ± 26.6	116.3 ± 55.0	**<0.001**
Total Cholesterol (mg/dL)	155.9 ± 28.1	234.5 ± 34.0	**<0.001**
Triglycerides (mg/dL)	143.3 ± 78.8	155.6 ± 75.4	0.410
HDL-c (mg/dL)	43.9 ± 9.3	49.1 ± 10.6	**0.004**

Data displayed: mean ± standard deviation. *p* value by Mann–Whitney rank sum test. Bold numbers indicate *p* < 0.05.

**Table 6:** Multivariate linear regression model predicting LDL-c levels.

Predictor	B Coefficient (95% IC)	*p* Value
Age (years)	0.2667 (0.0312, 0.5022)	**0.027**
Gender (female)	6.4783 (−1.7974, 14.7542)	0.125
BMI (kg/m^2^)	0.5500 (0.1808, 0.9191)	**0.004**
Glucose (mg/dL)	0.2834 (0.1824, 0.3843)	**<0.001**
Triglycerides (mg/dL)	0.0597 (0.0189, 0.1005)	**0.004**
GRS	19.4725 (1.7701, 37.1749)	**0.031**
WHR	245.9810 (71.9502, 420.0117)	**0.006**
GRS × WHR	−26.5307 (−47.6621, −5.3993)	**0.014**
Adj. R-squared	0.1253	**<0.001**

Bold numbers indicate *p* < 0.05.

## Data Availability

The data presented in this study are available on request from the corresponding author. Data are not publicly available due to privacy reasons and because the data are part of an ongoing study.
